# Semi-Automatic Integrated Segmentation Approaches and Contour Extraction Applied to Computed Tomography Scan Images

**DOI:** 10.1155/2008/759354

**Published:** 2008-10-27

**Authors:** B. Dhalila S. Y. Khoodoruth, Harry C. S. Rughooputh, Wilfrid Lefer

**Affiliations:** ^1^Department of Computer Science, University of Pau and Pays de l'Adour, 64012 PauCedex, France; ^2^Department of Electrical & Electronic Engineering, University of Mauritius, Reduit, Mauritius

## Abstract

We propose to segment two-dimensional CT scans traumatic brain injuries with various methods. These methods are hybrid, feature extraction, level sets, region growing, and watershed which are analysed based upon their parametric and nonparametric arguments. The pixel intensities, gradient magnitude, affinity map, and catchment basins of these methods are validated based upon various constraints evaluations. In this article, we also develop a new methodology for a computational pipeline that uses bilateral filtering, diffusion properties, watershed, and filtering with mathematical morphology operators for the contour extraction of the lesion in the feature available based mainly on the gradient function. The evaluations of the classification of these lesions are very briefly outlined in this context and are being undertaken by pattern recognition in another paper work.

## 1. INTRODUCTION

Segmentation is crucial for image analysis. The segmented features can be used for presurgery planification or diagnostic purposes as referred to Ciofolo and Barillot [1]. For instance, an irregular boundary of a segmented subdural haematoma in the parietal lobe indicates the severity of the traumatic brain injury as acute stage. Parametric Segmentation approaches such as feature extraction, hybrid, level-sets, watershed and region-growing are experimented. These evaluations are performed for traumatic brain injuries such as brain atropy, subdural hygroma, subdural haematoma, nonhaemorrhagic contrecoup contusion, and extracranial haematoma.

Parametric hybrid approaches such as deformable models, fuzzy connectedness, and fuzzy voronoi have been experimented, but the resultant output of the segmented pathological features is less efficient than those of the nonparametric segmentation approaches. In the case of the deformable models, the vector field lacks precision to identify the boundary of the feature. Whilst for the fuzzy connectedness, the binary template has generated quite effectively some results but not the exact representation of the segmented lesion. Any voronoi diagram is obtained by the fuzzy voronoi approach. The feature extraction approach such as hough transform 2D lines shows no effective result as the hough filter cannot compute the maxima in the hough map to define a geometry to locate the lesion.

The nonparametric segmentation approaches such as region growing approaches for confidence connected, connected threshold, neighbourhood connected are experimented.

Level-sets' approaches for threshold segmentation, fast marching, and shape detection mainly compute and analyse the motion of the feature under a velocity field which depends on the position, time, and geometry of the lesion as described in the work of Hong-Kai et al. [2]. Various results have been obtained with various ranges of constraints. The watershed-based segmentation approach is applied where the gradient magnitude of intensities of the pixels in the feature undergoes a transform.

The contour extraction of the brain atrophy, subdural haematoma, nonhemorrhagic contusion, and subdural hygroma is performed by a computational pipeline from the raw CT scans.

Section 2 is the state of the art related to segmentation. Section 3 describes the various approaches of segmentation applied to the traumatic brain injuries with the validations and evaluations of constraints. Section 4 describes our approach for the computational pipeline for the contour extraction. Section 5 concludes part of the work accomplished.

## 2. STATE OF THE ART

Basically, segmentation is the partitioning of the image into nonoverlapping constituent regions which are homogeneous with respect to some characteristics such as intensity or texture as described in the work of Bazin and Pham [3]. The identification of the pixels for a particular lesion is inherently built-in the segmentation method which can further allow for a pixel classification and labelling of the structure.

The dimensionality of the image is a crucial factor to be considered before segmentation process because image intensities are independent of the image domain. As described in the work of Mahrous et al. [4], the original vector field can be replaced by a derived segmented data set. The derived set is used to produce separating surfaces in the vector field whereas the CT scans are used for single scalar field.

Contour based surface extraction and isosurface extraction models have been considered for the purpose of implicit segmentation which is based on intensity threshold where points are classified as either greater or less than a given intensity as referred to Pham et al. [5]. The above is compared to an explicit segmentation using deformable models by reffering to the works of Giachetti and Zanetti [6], Colliot et al. [7]. The purpose is not to modify the image surface by an image vector and an internal image force.

Regarding the work of Hassouna et al. [8], these slight ambiguities have been encountered by the intensity regions based on the Hounsfield scale for CT scans. The purpose is to identify the grayscale range within the lesions. As stated in the works of Ritter et al. [9] and Heuberger et al. [10], the threshold segmentation is efficient for bone segmentation from CT scans. Since bone tissue attenuates significantly more x-rays during acquisition. Therefore, these attenuations are represented by much higher values on the Hounsfield scale compared to soft tissues. Weber et al. [11] developed an algorithm to modify a segmentation based on visual examination and obtained additional information about incorrectly segmented objects.

## 3. SEGMENTATION

The segmentation of these features must be considered based upon either a geometric approach or a diffusion approach or a fuzzy segmentation with a statistical approach as stated by Petersch et al., Vidal et al., and John et al. [12–14]. In the case of nonparametric level set segmentation methods, a geometric approach such as the distance function with a fast sweeping function is considered where the distance transform is calculated from the number of pixels as referred to Sifakis and Tziritas [15]. These segmentation approaches with different functionalities are experimented using open-source software insight toolkit [16] and a viewer.

### 3.1. Methodology

#### 3.1.1. Level sets

Based upon the excellent review of Osher and Fedkiw [17] on level sets, the surface Γ is represented as the zero isocontour of a scalar function (*x*), that is,

(1) Γ = x : ϕ(x) = 0,

which represents the curve or boundary of the feature.

The various approaches such as fast marching, threshold, and shape detection use basically the algorithmic representation of the level set procedure as described in Algorithm 1.

**Fast marching approach**

The FastMarchingImageFilter is used in the Reinitialize LevelSetImageFilter object to create a signed distance function from the zero level set as referred to [16].

**Figure 1:**
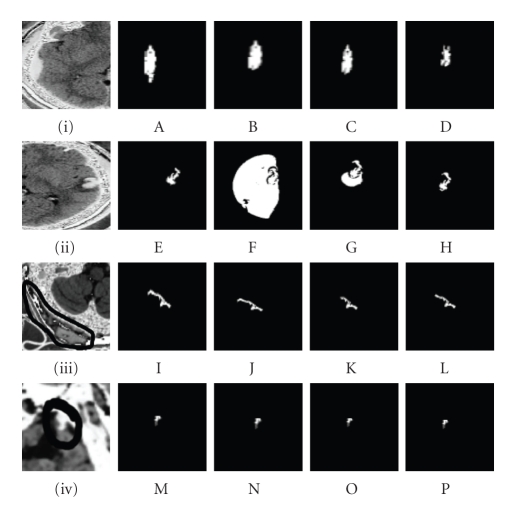
(A, E, I, M) *α* = −0.0005, *β* = 0.0005, sigma = 0.0005, (B, F, J, N) *α* = −0.0015, beta = 0.0025, *σ* = 0.0125, (C, G, K, O) *α* = −0.375, *β* = 0.2255, *σ* = 0.0225, (D, H, L, P) *α* = −0.575, *β* = 4.00, *σ* = 0.0325 are (i) values set for subdural haematoma, (ii) contrecoup contusion, (iii) extracranial haematoma, and (iv) brain atrophy by fast marching.

The lower threshold is set to a default value of 0.0 and the upper threshold defines the time snapshot which is taken from the time crossing map which is set to 85. The CurvatureAnisotropicDiffusionImageFilter requires TimeStep, NumberOfIterations, and ConductanceParameter are set as 0.125, 5, and 9 so as to detect the boundary of the structures. The SigmoidImageFilter class requires two parameters, alpha and beta, to define the linear transformation to the sigmoid argument as illustrated in Figures 1(E), 1(F), 1(G), 1(H) or reduction at edges as illustrated in Figures 1(A), 1(B), 1(C), 1(D).

By increasing the value of *β* to 4.00 and decreasing the value *α* to −0.575 as referred to in Table 1, the size of the feature gets smaller as illustrated in Figures 1(A), 1(B), 1(C), 1(D) and expands in Figures 1(E), 1(F), 1(G), 1(H). The shape of the feature gets distorted as the value of *σ* increases to 0.325. The threshold is generated to the time crossing map so as to build the time solution surface Γ of the white matter one grid point at a time where the feature is situated. Though the time is found for one grid point, but gets distorted for other nearby values in the features of Figures 1(i) and 1(ii).

The features of Figures 1(iii) and 1(iv) as mentioned in Table 1 show no variation during segmentation by decreasing or increasing the values of the constraints. The edges of the segmented features as illustrated in Figures 1(I), 1(J), 1(K), 1(L) are almost identical. Figures 1(M), 1(N), 1(O), 1(P) illustrate almost identical features. Since the extracranial haematoma is outside the skull and has a homogeneous tissues density throughout resulting in no variation of the gradient intensities, the brain atrophy is at the rim of the right frontal lobe of the skull where the variation of gradient intensities is almost stagnant. Consequently, the most crucial factor in these features (iii) and (iv) is the time crossing map, which is set at 0.125 due to the coil rotation inside the CT scanner.

The fast marching level set segmentation is deduced to be the most appropriate feature extraction for the extracranial haematoma and the brain atropy.

The extracranial haematoma is homogeneously hyperdense, sharply marginated, and consists of solid blood clots found on the outside border of the skull as referred to [18]. The pixel intensities within this feature are constant throughout. So, the gradient magnitude intensities computed do not vary though the *σ* values are within a range of 0.0005 to 0.325, the *α* values are within a range of −0.0005 to −0.575, and the *β* values are within a range of 0.0005 to 4.00. This implies that the sigmoid member class filter has no effect on the time solution surface Γ which is the zero level set representing the contour of the feature. The TimeStep of the scanner set at 0.125 and the number of iterations are crucial parameters to seek the proper edge of the feature.

**Threshold approach**

**Figure 2:**
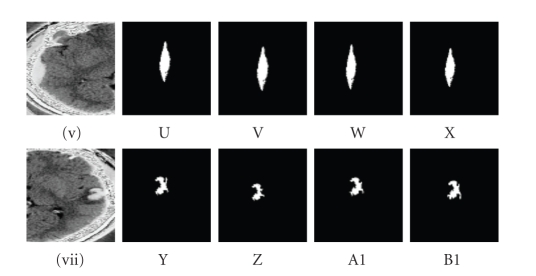
(U, Y) distance = 4, lower = 150, upper = 175, (V, Z) distance = 5, lower = 155, upper = 185, (W, A1) distance = 6, lower = 160, upper = 195, (X, B1) distance = 7, lower = 175, upper = 205 are (vi) values set for the subdural haematoma and (vii) contrecoup contusion by threshold approach.

A range of intensity values are defined for the feature in the threshold level set segmentation. A propagation term is applied on the level set equation for the intensity range as referred to [16] and illustrated in Figure 2.

The initial level set generates the distance map, and a distance function is computed by applying the lower and upper threshold. In case, the lower threshold is less than 150, as referred to in Table 2, and the higher threshold is greater than 205, the range of intensities for the feature does not determine an initial surface for the gradient flow resulting in no segmentation.

Values below 4 or greater than 7 for the distance map result in a reduction or expansion of the initial contour, the zero level set of the feature as the surrounding region is white matter of homogeneous tissues density. There is very slight change of segmented feature as illustrated in Figures 2(U), 2(V), 2(W), 2(X) and Figures 2(Y), 2(Z), 2(A1), 2(B1) by applying the range of 4 to 7 for the distance map.

The threshold level set segmentation is the appropriate procedure for segmenting the subdural haematoma and the nonhemorrhagic contusion.

Since the subdural haematoma is in an acute stage, it is hyperdense because of the attenuating properties of the haemoglobin molecules found in the blood clots. The stage of density takes weeks before being isodense. The lower and upper limits of the thresholds are set at 140 and 205 so as to consider only gradient magnitude intensities within the feature forbidding surrounding regions which can be hypodense or isodense. By lowering the limit of the threshold, lower gradient magnitude densities are acquired for which the distance function does not clearly mark the edge of the feature. The hyperdense area of the feature can be clearly demarcated by the proper threshold setting. When the threshold is increased to the corresponding gradient magnitude densities applied to the distance function, a proper segmented feature is obtained without any distortion of the initial surface of the zero level set.

Whilst the nonhemorrhagic contrecoup contusion is an area of higher densities of tissues sorrounded by areas of lower densities of tissues, the varying range of the minimum and maximum of the threshold values is adjust to the varying range of gradient magnitude densities. Consequently, the initial zero level set surface is computed by the adjustments of the corresponding densities and distance map resulting in a proper feature extraction.

**Shape detection approach**

Finally in the shape detection level set segmentation method, fast marching level set segmentation is used to define the initial level set based upon a distance map as referred to [16].

**Figure 3:**
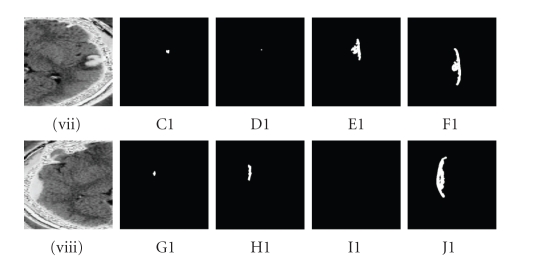
(C1, G1) distance = 3, *α* = −0.25, *σ* = 0.45, *β* = 1.85, propagation = 0.005, curvature = 0.9, (D1, H1) distance = 5, *α* = −0.35, *σ* = 0.65, *β* = 2.15, propagation = 0.035, curvature = 1.1, (E1, I1) distance = 7, *α* = −0.45, *σ* = 0.95, *β* = 2.9, propagation = 0.05, curvature = 1.3, (F1, J1) distance = 9, *α* = −0.55, *σ* = 1.1, *β* = 3.25, propagation = 0.07, curvature = 1.5 are values set for (viii) the contrecoup contusion and (ix) subdural haematoma by shape detection approach.

Though a wide range of parameter values are set for this approach, no exact segmented feature is obtained, as referred to in Table 3. The relative weightings of the propagation and curvature terms between these two parameters do not adjust to produce the shape boundaries of the feature. Consequently, zero level set leaks may have been produced through the feature's regions of low gradient along the boundaries of the pathological feature itself as illustrated in Figures 3(C1), 3(D1), 3(E10), 3(F1) and Figures 3(G1), 3(H1), 3(I1), 3(J1).

#### 3.1.2. Watershed

As described in the work of Grau et al. [19], and the excellent review of Roerdink and Meijster [20], the method is based on the topographical distance applied on these discrete two-dimensional medical images where basically the algorithmic representation is used.

**Figure 4:**
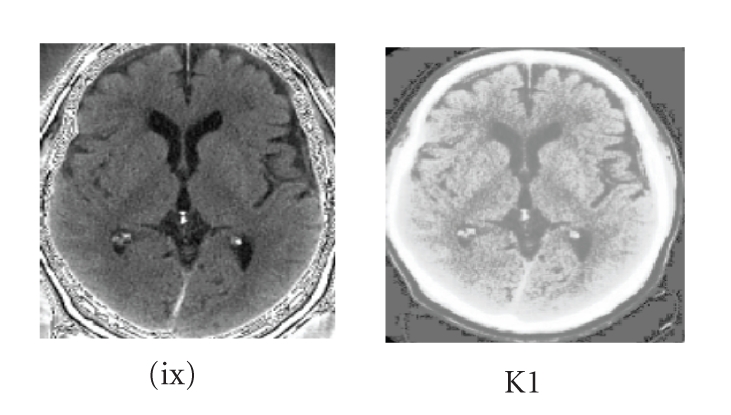
(column K1) Values set for the subdural hygroma (*x*) are conductance = 2.5, iterations = 11, lower = 0.015, scale level = 0.15 by watershed approach.

Two important parameters, threshold and level control the output of this watershed segmentation, as referred to in Table 5. The purpose of the threshold parameter is to set the absolute minimum height value. The level parameter controls the depth of metaphorical flooding of the image. Raising and lowering the level influence the number of segments in the basic segmentation that are merged to produce the final output. A level of 1.0 is analogous to flooding the image up to a depth that is 100 percent of the maximum value in the image. Level values of interest are typically low as illustrated in Figure 4, that is, less than about 0.40 since higher values will quickly undersegment the image.

The threshold parameter cannot exceed the value of 0.1 as illustrated in Figure 4(K1) to represent the gradient magnitude of intensities which are a percentage of the maximum depth of the feature so as to avoid oversegmentation.

#### 3.1.3. Region growing

In this approach, a seed is selected from the data set manually, and the algorithm engulfs the relevant region up to its boundary using some connectedness approach to the surrounding pixels as referred to [16]. Basically, the algorithmic representation of the region growing procedure is used for the confidence connected, connected threshold, and neighbourhood approaches.

**Figure 5:**
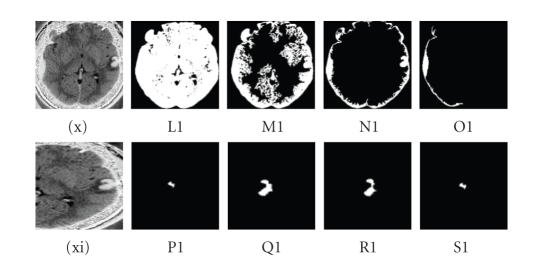
(L1) multiplier = 2.5, (M1) multiplier = 2.0, (N1) multiplier = 1.5, (O1) multiplier = 1.0, (P1) lower = 140, upper = 170, (Q1) lower = 150, upper = 180, (R1) lower = 155, upper = 190, (S1) lower = 160, upper = 195 are (xi) values set for the contrecoup contusion by confidence connected, for (xii) contrecoup contusion by neighbourhood connected.

The NumberOfIterations and TimeStep which are set as default values of 5 and 0.125 for 2D images, as referred to in Table 6. The itkConfidenceConnectedImageFilter class is used for the segmentation itself. This requires the definition of two parameters. The factor *f* which determines the extent of the range of intensities and the number of iterations specifies the homogeneity of the structure to be segmented. In this context, the multiplier factor is more important than the number of iterations, to avoid the region to engulf the entire image as illustrated in Figure 5(L1).

The range of values for the lower threshold and upper threshold varies between the minimum of 140 and maximum of 205. This allows for the usage of various pixel intensities to be identified for the computation of the gradient magnitude intensities due to its sensitivity to thresholds. As illustrated in Figure 5(P1), a minimum value of threshold 140 and maximum value of threshold 170 show a compression of the segmented feature, and the most exact representation of the segmented feature is illustrated in Figure 5(Q1) with a minimum of 150 and maximum of 180. No segmented feature expansion is obtained through this range, but still a compressed segmented feature is obtained with the minimum of 160 and maximum of 195 as illustrated in Figure 5(S1).

The nonhemorrhagic contusion is well segmented by the neighbourhood connected threshold approach. The varying range of minimum and maximum values of threshold of 140 and 205 allows the neighbouring filter to consider neighbouring pixels intensities instead of only the current pixel intensity. This is done by also accepting or rejecting small structures found inside or outside the lesion. Since the lesion is heterogeneous, the structuring element of the feature is well surrounded by the exact evaluation of gradient magnitude densities resulting in the exact segmented feature.

### 3.2. Constraints

The influence of each constraint has a direct impact on each lesion type, which is the most crucial part of the work undertaken. The segmented lesions types vary differently or constantly depending upon the range of the applied constraint. These variations are also assessed by the numerical evaluations and comparisons of the results.

#### 3.2.1. Numerical constraints evaluations

Herewith is the sets of constraints associated to the segmented lesion types after segmentation process.

**The sigma constraint**

The sigma “*σ*” constraint is efficient for the segmentation of the brain atrophy, extracranial haematoma, subdural haematoma and nonhemorrhagic contrecoup contusion. The edge of the segmented feature is kept linearly constant by the proper assignment of its numerical value.

Two ranges of values such as 0.0005 ≤ *σ* ≤ 0.325 and 0.45 ≤ *σ* ≤ 1.1 have been experimented. Negative and null values are forbidden resulting in leakages at the edges of the feature since the pixel type is of unsigned short type. Values less than 0.0005 result in expansion and values greater than 1.1 result in reduction of the segmented feature. According to Figure 1, the most appropriate result for the segmented feature lies in columns (B, F, J, N) and (C, G, K, O) within a range of 0.0125 to 0.0225.

As illustrated in Figure 1, these four lesions types are located either at the rim of the dura matter or just at the outline of the bone skull with a slight varying range of pixels intensities within each lesion type. The very slight range for an efficient segmented lesion is 0.0100 which explains the closeness and narrowness of the lesion to an edge.

**The alpha constraint**

The alpha “*α*” constraint is efficient for the segmentation of the brain atrophy, extracranial haematoma, subdural haematoma, and nonhemorrhagic contrecoup contusion. The edge of the segmented feature is kept linearly constant by the proper assignment of its numerical value till a threshold is applied to a time crossing map to the white matter.

Two ranges of values such as −0.0005 ≤ *α* ≤ −0.575 and −0.25 ≤ *α* ≤ −0.55 have been experimented. Negative and null values are used according to the requirements though the pixel is of unsigned short type. Values less than −0.0005 result in expansion, and values greater than −0.55 result in reduction of the segmented feature. According to Figure 1, the most appropriate result for the segmented feature lies in columns (B, F, J, N) and (C, G, K, O) within a range of −0.0015 to −0.375.

As illustrated in Figure 1, these four lesions types are located either at the rim of the dura matter or just at the outline of the bone skull with a slight varying range of pixels intensities within each lesion type. The very slight range for an efficient segmented lesion is −0.360 which explains the closeness and narrowness of the lesion to an edge.

**The beta constraint**

The beta “*β*” constraint is efficient for the segmentation of the brain atrophy, extracranial haematoma, subdural haematoma, and nonhemorrhagic contrecoup contusion. The edge of the segmented feature is kept linearly constant by the proper assignment of its numerical value.

Two ranges of values such as 0.0005 ≤ *β* ≤ 4.00 and 1.85 ≤ *β* ≤ 3.2 have been experimented. Negative and null values are forbidden resulting in leakages at the edges of the feature since the pixel type is of unsigned short type. Values less than 0.0005 result in expansion, and values greater than 4.00 result in reduction of the segmented feature. According to Figure 1, the most appropriate result for the segmented feature lies in columns (B, F, J, N) and (C, G, K, O) within a range of 0.0025 to 0.2255.

As illustrated in Figure 1, these four lesions types are located either at the rim of the dura matter or just at the outline of the bone skull with a slight varying range of pixels intensities within each lesion type. The very slight range for an efficient segmented lesion is 0.2230 which explains the closeness and narrowness of the lesion to an edge.

**The distance constraint**

The initial distance “initial distance” constraint is efficient for the segmentation of the subdural haematoma and nonhemorrhagic contrecoup contusion. The initial distance is the distance between the initial surface and the boundary of the feature which is assigned a numerical value.

Two ranges of values such as 4 ≤ initial distance ≤ 7 and 3 ≤ initial distance ≤ 9 have been experimented. Negative and null values are forbidden because this constraint is a crucial requirement to locate the lesion. Values less than 3 result in expansion, and values greater than 9 result in reduction of the segmented feature. According to Figure 2, the most appropriate result for the segmented feature lies in columns (W, A1) and (X, B1) within a range of 6 to 7.

As illustrated in Figure 2, these two lesion types are located at the rim of the dura matter with a slight varying range of pixels intensities within each lesion type. The very slight range for an efficient segmented lesion is 1 which explains the closeness and narrowness of the lesion to an edge.

**The lower threshold constraint**

The lower threshold “lower” constraint is efficient for the segmentation of the subdural haematoma, and nonhemorrhagic contrecoup contusion has been experimented. The lower threshold accesses current pixel intensities within the feature.

The ranges of values such as 150 ≤ lower ≤ 175 and 140 ≤ lower ≤ 175 have been experimented. Negative and null values are forbidden because if not the feature will not be selected at all. Values less than 150 result in expansion, and values greater than 175 result in reduction of the segmented feature. According to Figure 2, the most appropriate result for the segmented feature lies in columns (W, A1) and (X, B1) within a range of 160 and 175.

As illustrated in Figure 2, these two lesions types are located at the rim of the dura matter with a slight varying range of pixels intensities within each lesion type. The very slight range for an efficient segmented lesion is 15, which explains the closeness and narrowness of the lesion to an edge.

**The upper threshold constraint**

The upper threshold “upper” constraint is efficient for the segmentation of the brain atrophy, extracranial haematoma, subdural haematoma and nonhemorrhagic contrecoup contusion. The upper threshold accesses neighbouring pixel intensities of similar intensities within the feature.

Two ranges of values such as 175 ≤ upper ≤ 205 and 170 ≤ upper ≤ 205 have been experimented. Negative and null values are forbidden because if not the feature won't be selected at all. Values less than 140 result in expansion, and values greater than 175 result in reduction of the segmented feature. According to Figure 2, the most appropriate result for the segmented feature lies in columns (W, A1) and (X, B1) within a range of 195 to 205.

As illustrated in Figure 2, these two lesions types are located at the rim of the dura matter with a slight varying range of pixels intensities within each lesion type. The very slight range for an efficient segmented lesion is 10 which explains the closeness and narrowness of the lesion to an edge.

**The conductance constraint**

The conductance constraint is efficient for the segmentation of the subdural hygroma. This constraint helps for a proper definition of the boundary of the lesion.

The range of values such as 2 ≤ conductance ≤ 7 has been experimented. Negative and null values are forbidden because if not the feature's boundary will not be selected at all. Values less than 2 result in undersegmentation, and values greater than 7 result in oversegmentation of the feature. According to Figure 4, the most appropriate result for the segmented feature lies in column (K1) with a value of 2.5.

**The iterations constraint**

The iterations constraint is efficient for the segmentation of the brain atrophy, extracranial haematoma, subdural haematoma, and nonhemorrhagic contrecoup contusion. This constraint determines the mean and standard variance of the neighbouring pixels to be calculated and consequently the number of times to repeat the segmentation.

The range of values such as 10 ≤ iterations ≤ 20 has been experimented. Negative and null values are forbidden so as to obtain the best segmentation result. Values less than 0.0005 result in expansion, and values greater than 4.00 result in reduction of the segmented feature.

#### 3.2.2. Comparison of various results for constraints evaluations

**(i) The sigma constraint**

These values 0.0005, 0.0125, 0.0225, and 0.0325 are illustrated in Figure 1, and these values 0.45, 0.65, 0.95, and 1.1 are illustrated in Figure 3.

**(ii) The alpha constraint**

These values −0.0005, −0.0015, −0.375, and −0.575 are illustrated in Figure 1, and these values −0.25, −0.35, −0.45, and −0.55 are illustrated in Figure 3.

**(iii) The beta constraint**

These values 0.0005, 0.0025, 0.2255, and 4.00 are illustrated in Figure 1, and these values 1.85, 2.15, 2.9, and 3.25 are illustrated in Figure 3.

**(iv) The initial distance constraint**

These values 4, 5, 6, and 7 are illustrated in Figure 2, and these values 3, 5, 7, and 9 are illustrated in Figure 3.

**(v) The lower threshold constraint**

These values 150, 155, 160, 175 are illustrated in Figure 2, and these values 140, 150, 155, 160 are illustrated in Figure 5.

**(vi) The upper threshold constraint**

These values 175, 185, 195, 205 are illustrated in Figure 2, and these values 170, 180, 190, 195 are illustrated in Figure 5.

#### 3.2.3. Requirements

The traumatic brain injuries segmented are brain atrophy, subdural hygroma, subdural haematoma, nonhemorrhagic contrecoup contusion, and extracranial haematoma obtained from a set of 5 patients.

These axial CT scans obtained from Centre Hospitalier De Bayonne are of dimension 512 × 512 and thickness 2.5 mm varying in between 38 to 182 slices effected from different medical practitioners which have been converted into 256 × 256. These slices show varying levels of intensities depending on the intra and intervisual perception of these practitioners.

Before segmentation proceeds, these images are preprocessed by the bilateral image filter which is an edge preserving smoothing filter. Smoothing is performed by using domain and range neighbourhoods. Pixels that are close to a pixel in the image domain and similar to a pixel in the image range are used to calculate the filtered value as referred to Tomasi and Manduchi [21].

## 4. CONTOUR EXTRACTION

In our approach, the contour is a feature tracking of the lesions as referred to Loncaric et al., Deriche, and Roover et al. [22–24]. CT scans have a static scalar field of vision as mentioned in Sohn and Bajaj [25]. Diffusion properties have been applied in our computational pipeline preceded by smoothing and edge-preserving from a bilateral filter using open-source software insight Toolkit (ITK) [16]. The diffusion data enhances the properties of the components in these CT scan images as mentioned in Breen et al. [26] and Straka et al. [26, 27]. The gradient magnitude of the diffusion anisotropy is analysed by the watershed transform by graphing a function to create the merge tree. The gradient magnitude of the watershed pixels is transformed into the gradient magnitude of the diffusion anisotropy.

Mathematical morphology grayscale operators such as dilation and erosion use the merge tree of these connected flood levels of varying values. Each intersection node of the connected flood levels is used to create a graph. The higher values of the flood levels receive the maximum values of the dilation operator as referred to the work of Droogenbroeck [28], Droogenbroeck and Talbot [29], and Vardavoulia et al. [30] and the lower values of the flood levels receive the minimum values of the erosion operator. The nodes connect the intersection points which are linked so as to represent the contours corresponding to their features. Finally, some smoothing has been performed in a viewer for edge-preserving of the eroded output images.

### 4.1. Methodology

After the raw CT scans are preprocessed by the bilateral filter. The *h*(*x*) components of the output image move to the gradient anisotropic diffusion image filter. The anisotropic diffusion measures the diffusion properties of the water molecules in the cerebral tissues *C* where *D* is the diffusion coefficient:

(2) $$\begin{array}{ll} \frac{\delta C}{\delta t} & \;=\;\nabla \;\cdot \;(D\nabla C),\\ D & \;=\;\left(\begin{array}{cc} D_{xx} & D_{xy}\\ D_{yx} & D_{yy} \end{array}\right). \end{array}$$

The invariants are the eigenvalues of diffusion *D* which are the roots of corresponding characteristic:

(3) $$\lambda^{3}\;-\;C_{1}\;\cdot \;\lambda^{2}\;+\;C_{2}\;\cdot \;\lambda \;-\;C_{3}\;=\;0,$$

with coefficients

(4) $$\begin{array}{ll} C_{1} & \;=\;\lambda_{1}\;+\;\lambda_{2}\;+\;\lambda_{3},\\ C_{2} & \;=\;\lambda_{1}\lambda_{2}\;+\;\lambda_{1}\lambda_{3}\;+\;\lambda_{2}\lambda_{3},\\ C_{3} & \;=\;\lambda_{1}\lambda_{2}\lambda_{3}, \end{array}$$

which are proportional to the sum of the radii and surface area of the pathological feature. The dataset is described as (*C*_1_, *C*_2_, *C*_3_) since *C*_*i*_ are the coefficients of the characteristic equation. Considering the dimensionless combination *C*_1_ *C*_2_/*C*_3_, it becomes

(5) $$\frac{C_{1}C_{2}}{C_{3}}\;=\;3\;+\;\frac{\lambda_{2}\;+\;\lambda_{3}}{\lambda_{1}}\;+\;\frac{\lambda_{1}\;+\;\lambda_{3}}{\lambda_{2}}\;+\;\frac{\lambda_{1}\;+\;\lambda_{2}}{\lambda_{3}},$$

where a new dimensionless anisotropy measure is defined as

(6) $$C_{a}\;=\;\frac{1}{6}\left[\frac{C_{1}C_{2}}{C_{3}}\;-\;3\right]$$

for a linear, directional diffusion (*λ*_1_ ≫ *λ*_2_ ≈ *λ*_3_) is equal to

(7) $$C_{a}^{\text{limit}}\approx \frac{1}{3}\left[1\;+\;\frac{\lambda_{1}}{\lambda_{3}}\;+\;\frac{\lambda_{3}}{\lambda_{1}}\right].$$

Thus, *C*_*a*_ is always ∼*λ*_max_/*λ*_min_ and measures the gradient magnitude of the diffusion anisotropy.

The watershed transform is calculated on the absolute value of the gradient with high values at features' contours. Calculation of the watershed transform using arc weights in a graph allows the substitution of the usual gradient estimation by absolute differences of gray level calculated directionally during the flooding process to achieve a higher resolution. The lower slope is redefined as

(8) $$\text{LS}(p)\;=\;\underset{q\in N_{G}(p)\cup p}{\text{max}}\;\left(\frac{g(p,q)}{d(p,q)}\right),$$

where *g*(*p*, *q*) is a new function for the link (*p*, *q*). An equivalent function is calculated for the lower neighbours from

(9) $$\Gamma (p)\;=\;\{p^{\prime }\;\in \;N(p)\;|\;g(p,p^{\prime })\;=\;\underset{p^{\prime \prime }\in N_{G}(p)\cup p}{\text{max}}\;\;g(p,p^{\prime \prime })\}.$$

This condition allows the use of the directional diffusion anisotropy gradient magnitude value in substitution of the absolute value of the gradient resulting in

(10) $$\{p\;\in \;N,\;g(p,q)\;=\;g(p,C_{i})\},$$

where the neighbor space *N* is referred to as a set *Z* in which the component *q* of the function link is substituted by the component *C*_*i*_.

The discrete morphological gradient is

(11) δ(f) − ε(f).

The erosion and dilation functions by a gray-scale structuring element as referred to Droogenbroeck, and Darbon et al. [28, 31], *g* : *G*→*Z* are applied to the new links {*g*(*p*, *C*_*i*_)} such that

(12) ε(f) = (f ⊖ g)(x),

where

(13) $$\begin{array}{c} (f\;⊖\;g)(x)\;=\;\underset{y\in G}{\min}\;\{f(x\;+\;y)\;+\;g(y)\},\\ \delta (f)\;=\;(f\;⊕\;g)(x), \end{array}$$

where

(14) $$(f\;⊕\;g)(x)\;=\;\underset{y\in G,\;x-y\in F}{\max}\{f(x\;-\;y)\;+\;g(y)\},$$

respectively, where *x*, *y* ∈ *Z* ^2^ are the spatial coordinates. *F*, *G* ⊆ *Z* ^2^ are the domains of the grayscale image (function) and grayscale structuring element.

### 4.2. Program flowchart for contour extraction

**Figure 6:**
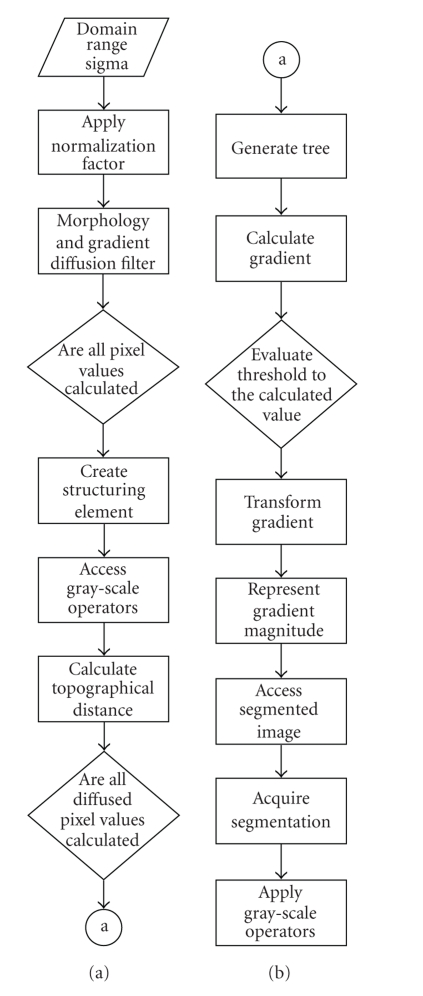
Program flowchart for contour extraction.

Let *f*(*x*) be the original grayscale image defined in the domain *D*_*i*_ and let *g*(*x*) be the output image as illustrated in Figure 6.

### 4.3. Implementation and results

**Figure 7:**
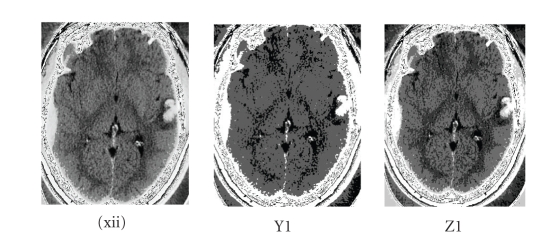
k-means classification.

The contour extraction traced out the exact feature as illustrated in Figure 7 and Table 6 of constraints.

### 4.4. Reasons for contour extraction technique compared to active contour model

The CT scans are from an “8-barrette” CT scanner. The removal of noise and filtering are a tedious task to get an optimized contrast of these CT images.

Since the active econtour geodesic/geometric models as referred to the work of Yoon et al. [32] are sensitive to these above fluctuations, the experimented technique has been implemented and preferred to active contour model because of the varying strength of the edges of these CT scans.

The speed at which the contours have been extracted by the experimented technique is fast compared to another model. Since the calculation of the speed of convergence with active contour model will require other parameters to be implemented.

## 5. CONCLUSIONS

We have presented a new methodology through the gradient calculation for the feature sets further enhanced by mathematical morphology grayscale operators. This computational pipeline technique is applied to CT scan images illustrating a subdural haemorrhage, a nonhemorrhagic contusion, a subdural hygroma, and a brain atrophy. In the case of the subdural hygroma, this technique demonstrates similar or superior performance to manual segmentation by experts. Without any doubt, the precision and accuracy of the segmented features in the contour tracking are reliable for diagnostic or presurgery purposes.

The deductions of the particular pathological feature extraction by particular segmentation procedures are mostly relevant in the selection process. The validation of the constraints settings of each segmentation procedure based upon the particular characteristics of trauma lesions is the determinant factors to be considered.

Finally, the substitution of one constituent of the link component in the gradient function of the watershed transform in the computational pipeline by the constituent of linear directional diffusion anisotropy gradient magnitude is one of the new methodology brought forward. The classification of these lesions by pattern recognition is tackled in another paper work.

## Figures and Tables

**Figure 8:**
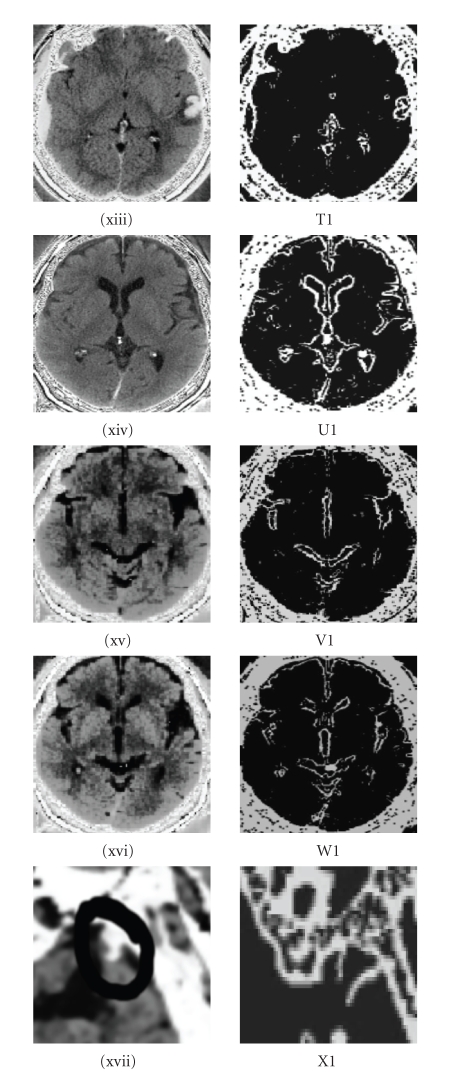
(T1) contour extraction of subdural haematoma and nonhemorrhagic contusion (xiii), (U1) contour extraction of subdural hygroma (xiv), slice 17, (V1) contour extraction of subdural hygroma (xv), slice 15, (W1)contour extraction of subdural hygroma (xvi), slice 16, (X1) contour extraction applied to brain atropy on left frontal lobe (xvii).

**Algorithm 1:**
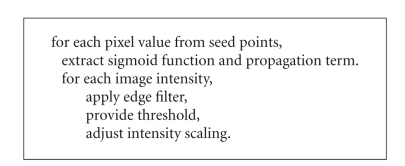
Algorithmic representation of level sets procedure.

**Algorithm 2:**
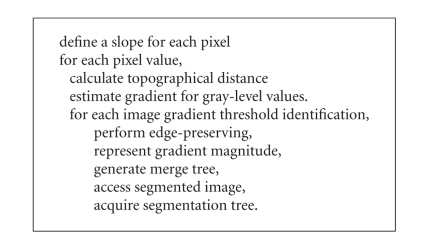
Algorithmic representation of watershed procedure.

**Algorithm 3:**
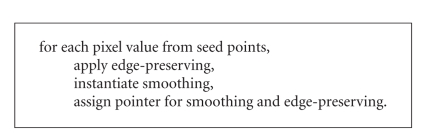
Algorithmic representation of region growing procedure.

**Table 1:** Internal constraints for fast marching level sets.

Fast marching	
Sigma	0.0005 ≤ *σ* ≤ 0.325
Alpha	−0.0005 ≥ *α* ≥ −0.575
Beta	0.0005 ≤ *β* ≤ 4.00

**Table 2:** Internal constraints for threshold level sets.

Threshold level set	
Initial distance	4 ≤ initial distance ≤ 7
Lower threshold	150 ≤ lower ≤ 175
Upper threshold	175 ≤ upper ≤ 205

**Table 3:** Internal constraints for shape detection level sets.

Shape detection	
Initial distance	3 ≤ initial distance ≤ 9
Sigma	0.45 ≤ *σ* ≤ 1.1
Alpha	−0.25 ≥ *α* ≥ −0.55
Beta	1.85 ≤ *β* ≤ 3.25
Propagation scaling	0.005 ≤ propagation ≤ 0.07
Curvature scaling	0.9 ≤ curvature ≤ 1.5

**Table 4:** Internal constraints for watershed.

Watershed	
Conductante term	2 ≤ conductance ≤ 7
Iterations	10 ≤ iterations ≤ 20
Lowerthreshold	0.001 ≤ Threshold ≤ 0.1
Output scale level	0.15 ≤ Scale level ≤ 0.30

**Table 5:** Internal constraints for region growing.

Confidence connected	
Multiplier	0.05 ≤ multiplier ≤ 2.5

Connected threshold

Lower threshold	150 ≤ lower ≤ 175
Upper threshold	175 ≤ upper ≤ 205

Neighbourhood

Lower threshold	140 ≤ lower ≤ 175
Upper threshold	170 ≤ upper ≤ 205

**Table 6:** Tables of required constraints for contour extraction of pathological features.

Computational pipeline	
Conductante term	2
Iterations	10
Lower threshold	0.05
Output scale level	0.15
Domain sigma	0.015
Range sigma	0.85
Time step	0.125

## APPENDIX

### CLASSIFICATION BY K-MEANS

The *k*-means model defines classes that represent statistical distribution of intensity values in the pixels as referred to [16].

The following results are for the subdural haematoma and contrecoup contusion as illustrated in Figure 7.

Figure 7, image(Y1): parameters are smoothing factor: 0, number of classes: 3, means: 14.8, 91.6, 134.9.

Result of estimated mean:

cluster[0] estimated mean: 33.6571,
cluster[1] estimated mean: 101.885,
cluster[2] estimated mean: 205.597.

Figure 7, image(Z1): parameters are smoothing factor: 1, number of classes: 5, means: 17.9, 112.6, 154.9, 182, 201.

Result of estimated mean:

cluster[0] estimated mean: 23.831,
cluster[1] estimated mean: 70.5066,
cluster[2] estimated mean: 114.158,
cluster[3] estimated mean: 190.176,
cluster[4] estimated mean: 232.982.

The estimated mean in the model estimated by the classifier.
